# Patterns of privilege: A total cohort analysis of admission and academic outcomes for Māori, Pacific and non-Māori non-Pacific health professional students

**DOI:** 10.1186/s12909-016-0782-2

**Published:** 2016-10-07

**Authors:** Erena Wikaire, Elana Curtis, Donna Cormack, Yannan Jiang, Louise McMillan, Rob Loto, Papaarangi Reid

**Affiliations:** 1 Te Kupenga Hauora Māori, Faculty of Medical and Health Sciences, University of Auckland, Private Bag 92015, Auckland, New Zealand; 2 Department of Statistics, Faculty of Science, University of Auckland, Private Bag 92015, Auckland, New Zealand

**Keywords:** Indigenous, Workforce development, Ethnic minority, Health professional, Tertiary education, Academic success, Māori, Pacific

## Abstract

**Background:**

Tertiary institutions are struggling to ensure equitable academic outcomes for indigenous and ethnic minority students in health professional study. This demonstrates disadvantaging of ethnic minority student groups (whereby Indigenous and ethnic minority students consistently achieve academic outcomes at a lower level when compared to non-ethnic minority students) whilst privileging non-ethnic minority students and has important implications for health workforce and health equity priorities. Understanding the reasons for academic inequities is important to improve institutional performance. This study explores factors that impact on academic success for health professional students by ethnic group.

**Methods:**

Kaupapa Māori methodology was used to analyse data for 2686 health professional students at the University of Auckland in 2002–2012. Data were summarised for admission variables: school decile, Rank Score, subject credits, Auckland school, type of admission, and bridging programme; and academic outcomes: first-year grade point average (GPA), first-year passed all courses, year 2 – 4 programme GPA, graduated, graduated in the minimum time, and composite completion for Māori, Pacific, and non-Māori non-Pacific (nMnP) students. Statistical tests were used to identify significant differences between the three ethnic groupings.

**Results:**

Māori and Pacific students were more likely to attend low decile schools (27 % Māori, 33 % Pacific vs. 5 % nMnP, *p* < 0.01); complete bridging foundation programmes (43 % Māori, 50 % Pacific vs. 5 % nMnP, *p* < 0.01), and received lower secondary school results (Rank Score 197 Māori, 178 Pacific vs. 231 nMnP, *p* < 0.01) when compared with nMnP students. Patterns of privilege were seen across all academic outcomes, whereby nMnP students achieved higher first year GPA (3.6 Māori, 2.8 Pacific vs. 4.7 nMnP, *p* < 0.01); were more likely to pass all first year courses (61 % Māori, 41 % Pacific vs. 78 % nMnP, *p* < 0.01); to graduate from intended programme (66 % Māori, 69 % Pacific vs. 78 % nMnP, *p* < 0.01); and to achieve optimal completion (9 % Māori, 2 % Pacific vs. 20 % nMnP, *p* < 0.01) when compared to Māori and Pacific students.

**Conclusions:**

To meet health workforce and health equity goals, tertiary institution staff should understand the realities and challenges faced by Māori and Pacific students and ensure programme delivery meets the unique needs of these students. Ethnic disparities in academic outcomes show patterns of privilege and should be alarming to tertiary institutions. If institutions are serious about achieving equitable outcomes for Māori and Pacific students, major institutional changes are necessary that ensure the unique needs of Māori and Pacific students are met.

## Background

In New Zealand (NZ), ongoing patterns of health inequities for Māori (the indigenous peoples of New Zealand) and Tagata Pasifika (a heterogeneous composite of peoples with Pacific nation ancestry born and living in New Zealand) when compared to non-Māori non-Pacific peoples need to be addressed [1–3]. In 2013, Māori life expectancy was 73.0 years for Male and 77.1 years for Female compared to 80.3 years and 83.9 years for non-Māori Male and Female groups respectively [3]. In 2006, life expectancy was 6.7 years less for Pacific Males and 6.1 years less for Pacific Females compared to the total NZ population [4].

Key to addressing these health inequities is a health sector that is able to deliver culturally appropriate, relevant, safe and effective health care [5, 6]. This not only includes a culturally competent health workforce, but also requires building a larger capacity of indigenous and ethnic minority health professionals working across the health sector. In New Zealand, there is a critical shortage of Māori and Pacific health professionals [7–11]. Despite making up 15.6 % of the NZ population [3], in 2009 Māori made up 3 % of doctors, 6 % of nurses, 2 % of pharmacists and 5 % of dentists [12]. Similarly, Pacific peoples make up 7.4 % of the NZ population, 1 % of doctors, 0.2 % of pharmacists, 0.6 % of dentists and 2.2 % of nurses [13–15]. Under-representation of indigenous and ethnic minority peoples within health professions limits health sector ability to provide a culturally safe, competent and appropriate workforce that meets the diverse needs of the community it serves [10, 16].

Tertiary institutions that offer health professional training play a key role in supporting Māori and Pacific health workforce development [17] and thereby contributing to addressing Māori and Pacific health needs. Similar challenges are seen internationally whereby tertiary institutions aim to admit and successfully graduate a diverse student body [18]. However, there is evidence that tertiary institutions are failing to achieve equitable academic outcomes for indigenous and ethnic minority students [19, 20], with ongoing trends of underrepresentation of indigenous and ethnic minority students participating in and graduating from tertiary programmes. This is particularly concerning for health professional programmes that aim to recruit, retain and graduate more workforce-ready indigenous and ethnic minority health professionals [8, 11, 18]. In New Zealand, the Faculty of Medical and Health Sciences (FMHS) at the University of Auckland (UoA) offers undergraduate degree-level programmes in health sciences, nursing, pharmacy, medicine and optometry^1^. Whilst the FMHS makes a commitment to Māori and Pacific health workforce development through its Vision 20:20 initiative [17], first year bachelor course completion rates in 2014 were lower for Māori (76.8 %) and Pacific (61.8 %) students when compared to the total cohort (82.8 %) [21]. Understanding the reasons for inequities between ethnic groups is important to monitor institutional performance against equity targets and contribute to developing Māori and Pacific student support initiatives.

Māori and Pacific student support initiatives aim to understand and address the multiple reasons for inequities in academic outcomes in health professional study between ethnic groups; however, information specific to Māori and Pacific students is limited [2, 22, 23]. Some research has identified a broad mix of pre-tertiary, admission and early academic factors as helping or hindering academic success for Māori and Pacific students in tertiary health programmes. For example, pre-tertiary factors such as academic preparation (including secondary school academic achievement, exposure to science subjects, meeting tertiary admission prerequisites, and having clear career goals), socioeconomic status, availability of role models and mentors, whānau (family) support, work/life balance, access to childcare, financial support, clear career information, support systems, support to transition and first year academic results and environments [2, 10, 23–26]. These factors align with international research findings for other indigenous and ethnic minority health students [22, 27–30]. However, the majority of international literature in this area has tended to focus on total (predominantly white) student cohorts; describing ethnicity as a predictor variable rather than carrying out separate analysis for each ethnic group separately [31–33] and it is therefore difficult to generalise their findings to both a New Zealand and Māori/Pacific context. What New Zealand based research is available focuses predominantly on Māori students, and although some information specific to Pacific students is available, it would be mutually beneficial to increase what is known for Māori and Pacific student cohorts, both combined and separately. In addition, quantitative analyses in some studies have been limited by small numbers of enrolled students from ethnic minority groups and a lack of direct comparison between ethnic groups. Alongside the development of Vision 20:20, Māori and Pacific cohort numbers within the FMHS have increased substantially over the last 40+ years; subsequently providing sufficient data to allow detailed analysis by ethnic grouping from enrolment to graduation. Quantifying differences in exposure to helping and hindering factors that impact on academic success for different ethnic groups is expected to contribute to enhanced targeted support and findings from this study may be of interest to international audiences.

This project aimed to identify predictors of academic success for Māori and Pacific students within undergraduate tertiary health study at the University of Auckland by:

1. Providing a detailed description of Māori, Pacific and non-Māori non-Pacific student groupings at entry (admission) and exit (completion) from FMHS programmes using quantitative data.
2. Identifying differences in the distribution of pre-tertiary, admission and academic outcome variables between ethnic groupings.

## Methods

### Methodology

Kaupapa Māori Research (KMR) methodology was utilised [34]. KMR aligns with a Māori inquiry paradigm and provides the theoretical foundations on which to develop research processes [25]. This study operates by Kaupapa Māori principles such as: tino rangatiratanga (self-determination); taonga tuku iho (cultural aspirations); ako Māori (culturally preferred pedagogy); kia piki ake i ngā raruraru o te kainga (socio-economic mediation); whānau (extended family); kaupapa (collective philosophy); te reo me ōna tikanga; Te Tiriti o Waitangi (the Treaty of Waitangi); āta (growing respectful relationships); and whakapapa (relational framework to te ao Māori) [34–36]^2^. In the context of this research, Kaupapa Māori means:

- Operating from a Māori worldview that takes into account Māori realities (that is, acknowledging that health and educational outcomes for Māori and Pacific students (and peoples) are influenced by broad social, cultural, historical, political and economic contexts [35];
- Commitment to Māori leadership and control over the research;
- That the researcher/researched relationship is mutually beneficial;
- Commitment to Māori researcher professional development;
- Commitment to high quality ethnicity data
- That the research will be of benefit to Māori;
- That the research will investigate inequities between indigenous and other ethnic student groupings
- Explicit rejection of findings that suggest the culture or genetics of Māori or Pacific students are to blame for educational failures;
- That the research will critique structural power imbalances;
- That analysis and recommendations will require institutional change rather than requiring students to change themselves; and
- That interpretation and conclusions are mana-enhancing (i.e. empowering) for Māori participants and communities.

Kaupapa Māori in this study also means ensuring that the research is consistent with Pacific methodology [37] and acknowledges the similar effects of social impacts on health and education for Pacific peoples [13]. Pacific representation within the project team and advisory group acknowledges mutual expertise of these parties in Pacific health research and values Pacific knowledge and decision-making contribution [38]. The input of Pacific Health researchers and methodology of talanoa [37] allowed for meaningful exploration of and advocacy for issues amongst both Māori and Pacific students.

### Study design

This research was located within the Department of Māori Health (Te Kupenga Hauora Māori), FMHS, UoA, led and controlled by senior Māori health researchers, and overseen by an advisory group made up of Māori, Pacific, academic and administrative staff from the faculty. To ensure equal explanatory power for all ethnic groupings of interest, data from all students who enrolled in year two of the Bachelor of Health Sciences (BHSc), Bachelor of Nursing (BNurs), or Bachelor of Pharmacy (BPhar) programmes at the FMHS between 2002 and 2013 were included in this study [39]. Students who were currently enrolled for whom the minimum time required to complete their programme had not passed were excluded from the study. An observational study design was used. Secondary individual student demographic, admission and academic results data from 2001 – 2013 were sourced from *Student Services Online* (SSO) (the UoA web-based centralised student data management system).

### Ethnicity groupings

Self-identified student ethnicity was automatically categorised into Māori, Pacific, Asian, European/Pākehā, and Other ethnic groupings within SSO using a prioritisation protocol prior to sourcing of the data [40]. Prioritisation of Māori ethnicity (as the ethnicity of first priority) when multiple ethnicities are selected ensures accurate representation of Māori within analysis outcomes [1, 41]. However, those who identify with both Māori and Pacific ethnicity are only counted in the Māori group – therefore reducing the Pacific group numbers. This is not ideal in this research context and is acknowledged as a limitation. The Asian, Other and Pākehā/European categories were combined into one non-Māori non-Pacific comparator grouping given that student who self-identified as New Zealander were included within the Other category. Māori and Pacific^3^ categories remained separate given that different impacts on academic outcomes may be occurring for Pacific and Māori students.

### Conceptualisation of predictor variables

**Fig. 1:**
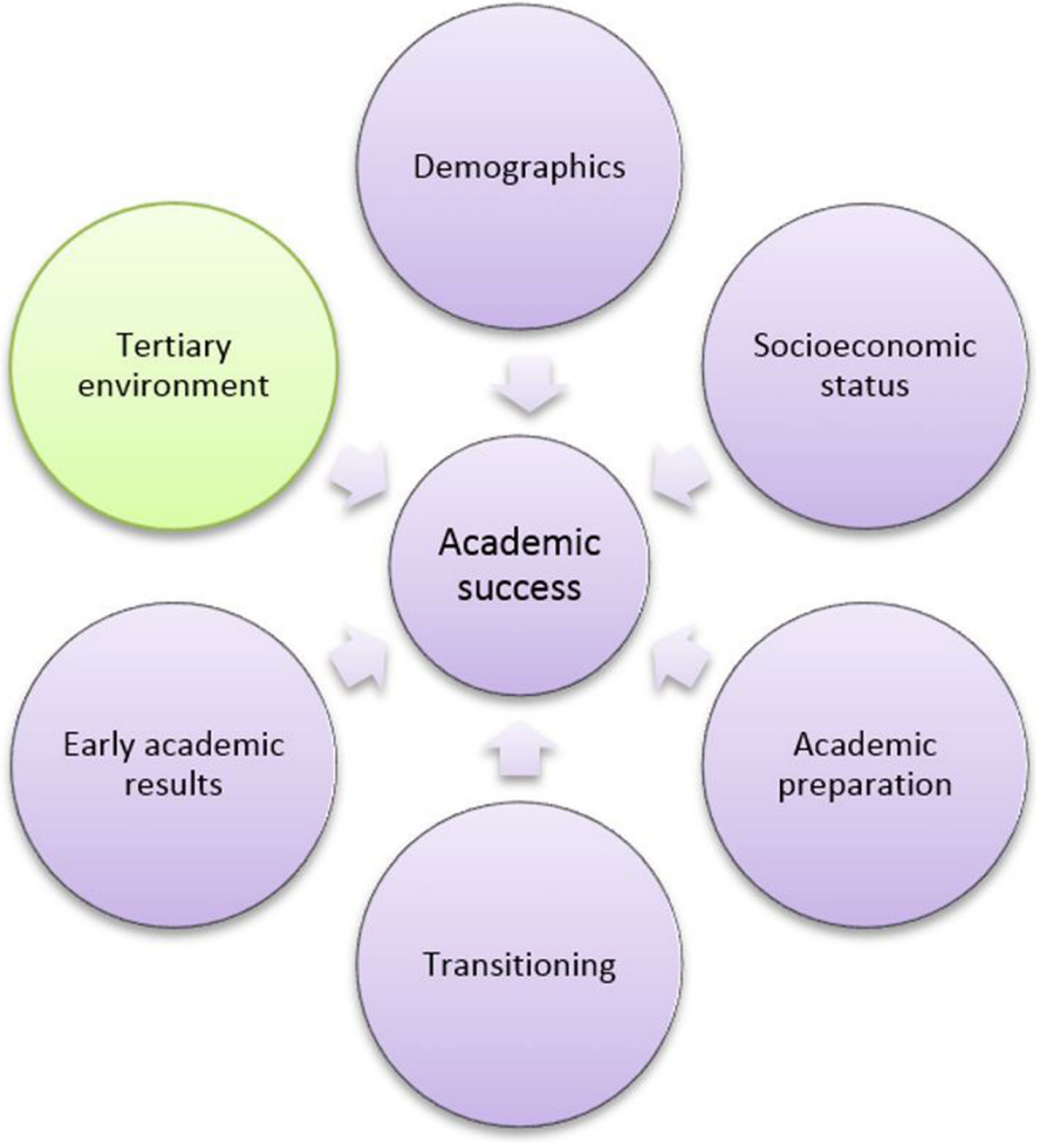
Conceptual model of predictors of academic success

A Kaupapa Māori theoretical approach to research methods was taken [42]. This included development of a ‘predictors of academic success’ model based on the Māori and Pacific health workforce development literature and experience within the FMHS context that foregrounds significant concepts that may impact on Māori and Pacific student success (Fig. 1). Key concepts in this model include: demographics (e.g. age, gender), socioeconomic status (e.g. economic status, poverty, housing, access to education), academic preparation (e.g. school results), transitioning (e.g. bridging foundation programmes, whānau support), early academic results (e.g. first year academic results) and the tertiary environment (e.g. curriculum). Each concept aims to group together a range of similar interacting factors that collectively may impact on student success. For example, the concept of academic preparation aims to include factors such as: academic achievement at school; exposure to science subjects; access to career information; and knowledge of required pre-requisites for entry, whereas the concept of early academic results aims to include factors such as: academic achievement in the first year of bachelor study; response to first year tertiary environments; and transitioning issues during this time. Available variables that most closely represented the concepts of importance as identified in the predictors of academic success model were derived from raw SSO data.

### Demographics

Demographic variables included *gender*, *age at admission* and *year of admission*. Gender is recorded as Male or Female; age was calculated as age in years on the 1^st^ March in the year of admission into stage two; year of admission is defined as the earliest year in which a student enrolled in a core stage two course for the BHSc, BNurs or BPharm programmes. Year of admission to stage two is presented for years 2002 – 2012 and is grouped into 2-year time periods to reduce risk of identification of students via enrolment numbers of less than 10.

### Socioeconomic status

Secondary school decile rating (1 – 10) was used as a measure of socioeconomic status in this study and was grouped into three categories: low (1 – 3), medium (4 – 7), and high (8 – 10) [32]. High decile represents schools within which a high proportion of students reside in areas of high socioeconomic status. Students who had attended school through correspondence (home schooled) or who had attended school outside of New Zealand (overseas) were coded as missing for this variable.

### Academic preparation

Academic preparation was measured using secondary school National Certificate in Educational Achievement (NCEA) Level 3 results (available from 2005 to 2013) that reflect FMHS bachelor degree entry requirements. Tertiary institutions encourage selection of and a high level of achievement in science, literacy and numeracy subjects (Table 1) at NCEA Level 3 for students wishing to pursue health professional study [10]. NCEA Rank Score^4^ represents an overall entry score and is presented as a continuous variable (0 – 320). Table A max was defined as the highest number of credits attained at Level 3 in one Table A subject (Table 1). Table B Maths max and Table B science max were defined as the highest number of credits attained at Level 3 in one Table B Maths subject or science subject respectively.

**Table 1:**
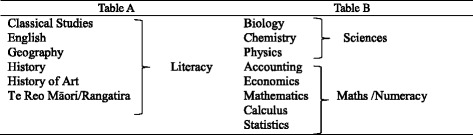
Table A and Table B approved NCEA Level 3 subjects for FMHS programme admission

### Transitioning

Transitioning that involved relocation was measured by identifying if the student had attended secondary school in the Auckland region (yes, no). Transitioning that involved varying pathways between secondary school and bachelor level admission were measured using type of admission (e.g. school leaver or alternative admission). School leaver (SL) was defined as enrolment in secondary school in the year immediately prior to enrolment in the first year of bachelor degree level study within FMHS. Alternative admission (AA) is defined as anyone who is not classified as a school leaver (including students who may have been transitioning directly from secondary school into a bridging foundation programme, and then on to first year bachelor level study).

Bridging foundation is conceptualised here as exposure to and completion of a UoA bridging foundation programme (yes, no) that aims to bridge the ‘gap’ between secondary and tertiary education contexts.

### Early academic results

Early academic results were measured using first year bachelor GPA (average ‘grade’ attained by each student in the first year of bachelor level study across eight courses) (0 – 9) and passing all courses (i.e. no ‘fail’ grades) in the first year of bachelor study (yes, no).

### Tertiary environment

There is a lack of measured variables representing tertiary environment factors (e.g. curriculum). Therefore, although the tertiary environment is conceptualised as impacting on student success in the ‘predictors of success model’, variables measuring these factors were not routinely collected within the SSO system.

### Academic outcome variables

Academic success was measured using ‘early academic’ and ‘programme’ outcomes.

### Early academic outcomes

Early academic outcome variables were conceptualised as being both ‘predicted by’ pre-tertiary factors and ‘predictors of’ longer term programme results, and are described above.

### Programme outcomes

Successful graduation from the intended programme ‘yes’ was defined as having graduated from the programme (BHSc, BNurs, BPhar) of original enrolment. Graduated in the minimum time (yes, no) is defined as completion of the FMHS programme in the minimum number of years (3 years for BHSc and BNurs, 4 years for BPharm). Year 2 – 4 programme GPA (0 – 9) was defined as the average grade achieved over all courses from year two until the final year of study.

### Programme composite outcome

Ideally, tertiary institutions aim to produce high calibre graduates with high employability. Thus, optimal completion in this context is defined as successful completion of the originally intended programme (yes), completion in the minimum time (yes), and achieving at least an A grade average (i.e. > = 6.6) across the entire programme. Sub-optimal completion with high grades was defined as successful completion of the originally intended programme (yes), completion in the minimum time (yes or no), and achieving at least a B grade average (i.e. > = 3.6) across the entire programme, and not already included in the optimal completion category. Sub-optimal completion with low grades was defined as successful completion of the originally intended programme (yes), completion in the minimum time (yes or no), and achieving at least a C grade average (i.e. 1 – 3.5) across the entire programme. All other students were categorised as non-completion (completion of intended programme (no) and completion in minimum time (no)) and represents those students who for varying reasons did not complete their intended programme.

### Analysis

Consistent with Kaupapa Māori research methodology, statistical analysis aimed to provide a detailed description of the characteristics of all three student ethnic groupings separately, particularly aiming to describe in detail all variables for the Māori and Pacific student groupings. In addition, Kaupapa Māori research aims to foreground inequities that may exist between ethnic minority student groupings (Māori and Pacific) and student ethnic groupings that make up the majority of the student cohort. Accordingly, statistical analysis comparisons were made between Māori and non-Māori non-Pacific student groupings and also between Pacific and non-Māori non-Pacific student groupings. Comparisons were not made between Māori and Pacific student groupings given that both groupings are considered to be of ethnic minority in this context.

Descriptive information was provided for all variables for each of the Māori, Pacific and non-Māori non-Pacific student cohorts as well as overall. Continuous variables were summarised as mean and standard deviation (SD). Categorical variables were described as frequency (n) and percentage (%). Distribution of the data between ethnic groupings was reviewed and tested, with the non-Māori non-Pacific students as the reference. For continuous variables, the analysis of variance model was used with pair-wise comparisons on the group means between Māori/Pacific students and non-Māori non-Pacific students. For categorical variables, the Chi-square test was used to compare the distribution of categories between groups. Statistical analysis was conducted using SAS version 9.4 (SAS Institute Inc., Cary, NC, USA). All statistical tests were two-sided at a 5 % significance level.

Ethics approval for this project was granted by the University of Auckland Human Participants Ethics Committee (Ref 8110). As per ethics protocols, written informed consent was not required for this research project due to the use of secondary administrative data sources. All secondary data obtained from these datasets were de-identified by an independent research member with no student contact or teaching responsibilities and data analysis occurred via a coding system.

## Results

### Demographics

A total of 2686 students were included in this study (Table 2). Non-Māori non-Pacific students made up the majority of the student cohort (84.8 %, *n* = 2279), followed by Pacific (9.6 %, *n* = 257) and Māori students (5.6 %, *n* = 150). The mean age for the total cohort at admission to year 2 of FMHS programmes was 20 years, with Māori students being slightly older compared to nMnP students (21.3 vs. 20.5 years old, *p* = 0.0061) (Table 2).

**Table 2:** Demographic and predictor variables for Māori, Pacific and non-Māori non-Pacific students

Demographic and admission variables	Ethnic grouping									
	Māori (*n* = 150)			Pacific (*n* = 257)			nMnP (*n* = 2279)		Total (*n* = 2686)	
*Categorical variables*	*n*	*%*	*p value*	*n*	*%*	*p value*	*n*	*%*	*n*	*%*
Gender			0.0944			0.0105		ref.		
Female	108	72.0		182	70.8		1775	77.9	2065	76.9
Male	42	28.0		75	29.2		504	22.1	621	23.1
Year of admission (2^nd^ yr.)^b^			–			–				
2002–3	25	16.7		28	10.9		320	14.0	373	13.9
2004–5	27	18.0		43	16.7		375	16.4	445	16.6
2006–7	23	15.3		54	21.0		428	18.8	505	18.8
2008–9	24	16.0		50	19.4		464	20.3	538	20.0
2010–11	34	22.7		53	20.6		502	22.0	589	21.9
2012	17	11.3		29	11.3		190	8.3	236	8.8
School Decile			<0.0001			<0.0001		ref.		
High (8–10)	54	36.0		51	19.8		1275	55.9	1380	51.4
Medium (4–7)	47	31.3		87	33.9		650	28.5	784	29.2
Low (1–3)	41	27.3		85	33.1		125	5.5	251	9.3
*Missing*	8	5.3		34	13.2		229	10.0	271	10.1
Auckland School			<0.0001			<0.0001		ref.		
No	63	42.0		12	4.7		320	14.0	395	14.7
Yes	81	54.0		211	82.1		1731	76.0	2023	75.3
*Missing*	6	4.0		34	13.2		228	10.0	268	10.0
Type of admission (1^st^ yr.)			<0.0001			<0.0001		ref.		
Alternative admission	77	51.3		156	60.7		630	27.6	863	32.1
School Leaver	73	48.7		101	39.3		1649	72.4	1823	67.9
Bridging programme			<0.0001			<0.0001		ref.		
No	85	56.7		129	50.2		2159	94.7	2373	88.3
Yes	65	43.3		128	49.8		120	5.3	313	11.6
Certificate in Health Sciences			–			–				
No	107	71.3		157	61.1		2279	100	2543	94.7
Yes	43	28.7		100	38.9		0	0	143	5.3
Programme enrolled^a^			–			–				
Health Sciences	99	66.0		185	72.0		696	30.5	980	36.5
Nursing	31	20.67		46	17.9		724	31.8	801	29.8
Pharmacy	26	17.33		39	15.2		917	40.2	982	36.6
*Continuous variables*	*Mean*	*SD*	*p value*	*Mean*	*SD*	*p value*	*Mean*	*SD*	*Mean*	*SD*
Age at admission (2nd yr.)	21.31	4.6	0.0061	20.8	3.7	0.1346	20.4	4.1	20.5	4.1
School results										
NCEA Rank Score	196.9	46.6	<0.0001	178.3	45.3	<0.0001	231.0	39.7	224.3	43.9
Table A Max	19.0	4.1	0.0008	18.4	5.1	<0.0001	20.7	3.8	20.4	4.0
Table B Max	22.8	6.6	0.0081	21.3	7.4	<0.0001	24.7	5.6	24.3	5.9
Table B Maths Max	21.5	6.1	0.0076	20.3	8.2	<0.0001	23.9	6.4	23.4	6.7
Table B Science Max	19.9	6.0	0.0157	16.9	5.5	<0.0001	21.4	4.4	20.9	4.8

^a^Students may have enrolled in more than one programme within the study duration; students enrolled in multiple programmes were double counted

^b^Although there were new enrolments in 2013, we have excluded current students and hence these students are not included in this data i.e. must have completed the minimum number of years required for their programme (i.e. three or four years). Results are presented for those variables that were tested for significant differences between ethnic groupings (the reference group of comparison is nMnP)

### Socioeconomic status

**Fig. 2:**
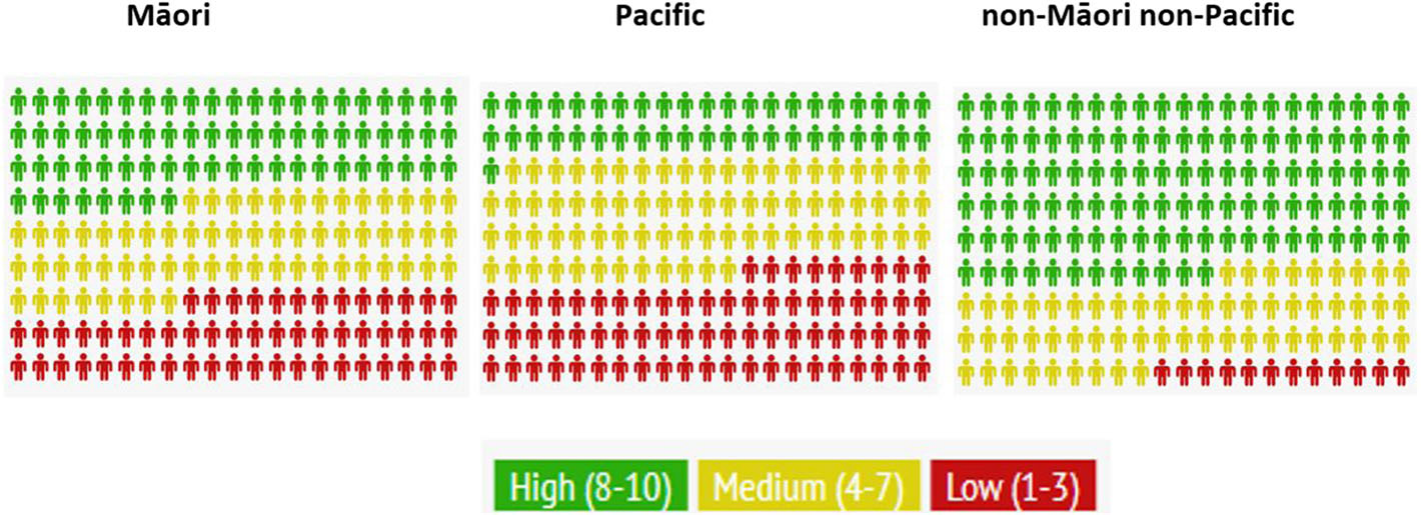
Infographic representing proportion of students by school decile and ethnic grouping

School decile was distributed significantly differently for both Māori and Pacific students when compared to the nMnP cohort (*p* < 0.0001) (Table 2, Fig. 2). For those students with school decile data, Fig. 2 provides an infographic depicting the distribution of Māori, Pacific and non-Māori non-Pacific students in this study cohort across the high, medium and low decile schools attended prior to admission. Of particular note is the low proportion of non-Māori non-Pacific students from low decile (red) schools (5 %) compared to much larger proportions of low decile (red) for Māori (27 %) and Pacific (33 %) student groups (Table 2).

### Academic preparation

The average NCEA Rank Score attained was 196.9 (SD 46.6) for Māori and 178.3 (SD 45.28) for Pacific students and both were significantly lower (*p* < 0.0001) than the average of 231 (SD 39.73) achieved by nMnP students^4^. The maximum number of credits achieved in Table A, Table B, Table B Science subjects and Table B Maths subjects on average were all significantly lower for both Māori and Pacific student cohorts when compared to nMnP students. One example shows that for Table B Science Max Māori students achieved on average 1.47 credits less (mean 19.9, SD 6.0, *p* = 0.0157), and Pacific students achieved on average 4.46 credits less (mean 16.9, SD 5.5, *p* < 0.0001) than nMnP students (mean 21.4, SD 4.4) (Table 2).

### Transitioning

A significantly higher proportion of Pacific students (82 %, *p* < 0.0001), and a significantly lower proportion of Māori students (54 %, *p* < 0.0001), had attended secondary school in Auckland compared with 76 % nMnP students^5^. A significantly higher proportion of nMnP students (72 %) had enrolled in bachelor level study as direct school leavers compared to less than half of Māori students (49 %, p < 0.0001) and just over one third of Pacific students (39 %, *p* < 0.0001). Similarly, significantly fewer nMnP students had completed a bridging foundation programme (5 %) compared to half of Pacific students (50 %, *p* < 0.0001) and two-fifths of Māori students (43 %, *p* < 0.0001). This is not surprising given the large proportion of Māori (*n* = 43, 29 %) and Pacific (*n* = 100, 39 %) students in this study who had completed the Certificate in Health Sciences, a Māori and Pacific specific bridging foundation programme within FMHS (Table 2).

### Early academic outcomes

**Fig. 3:**
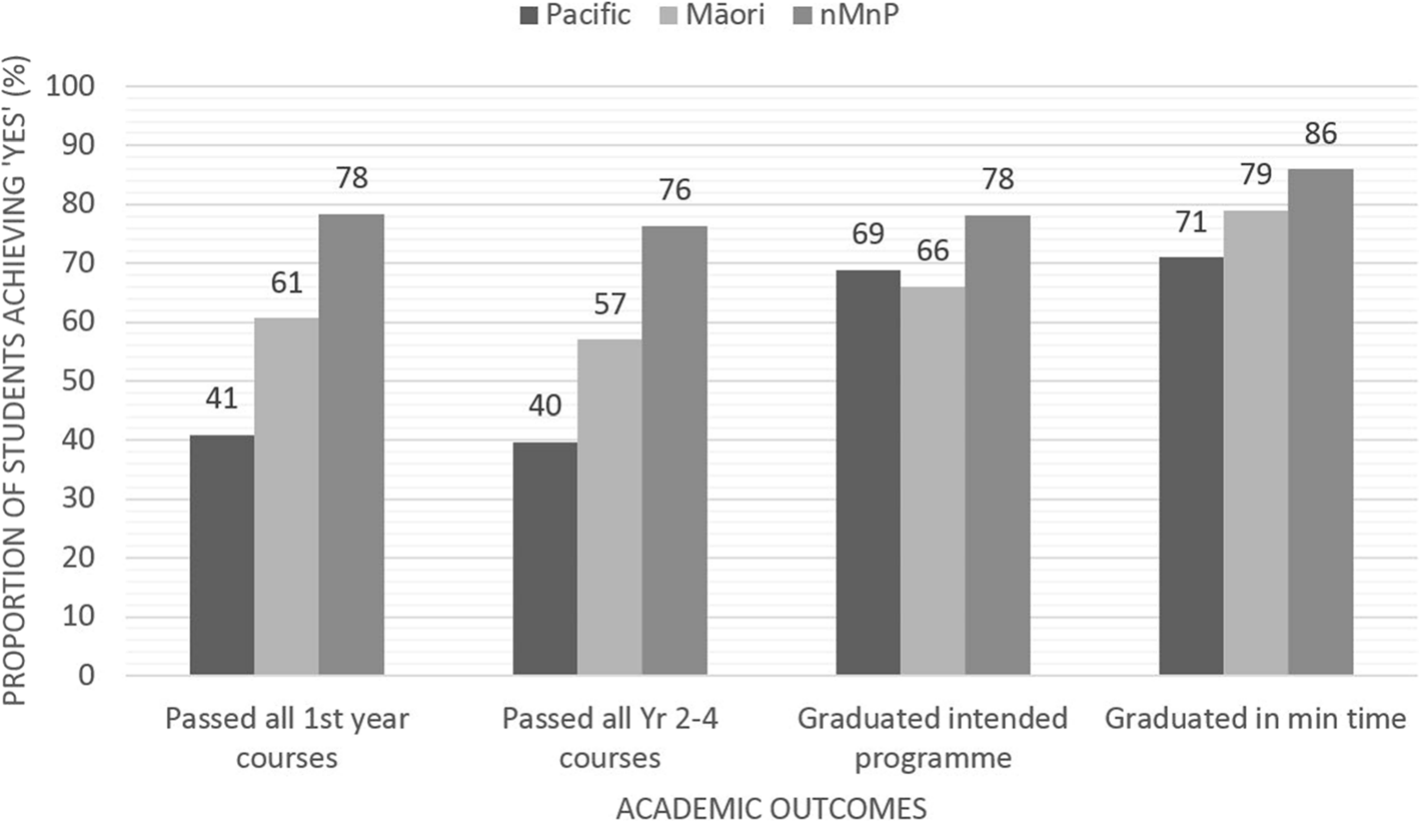
Proportion of Pacific, Māori and nMnP student groupings achieving (yes) categorical academic outcomes

**Fig. 4:**
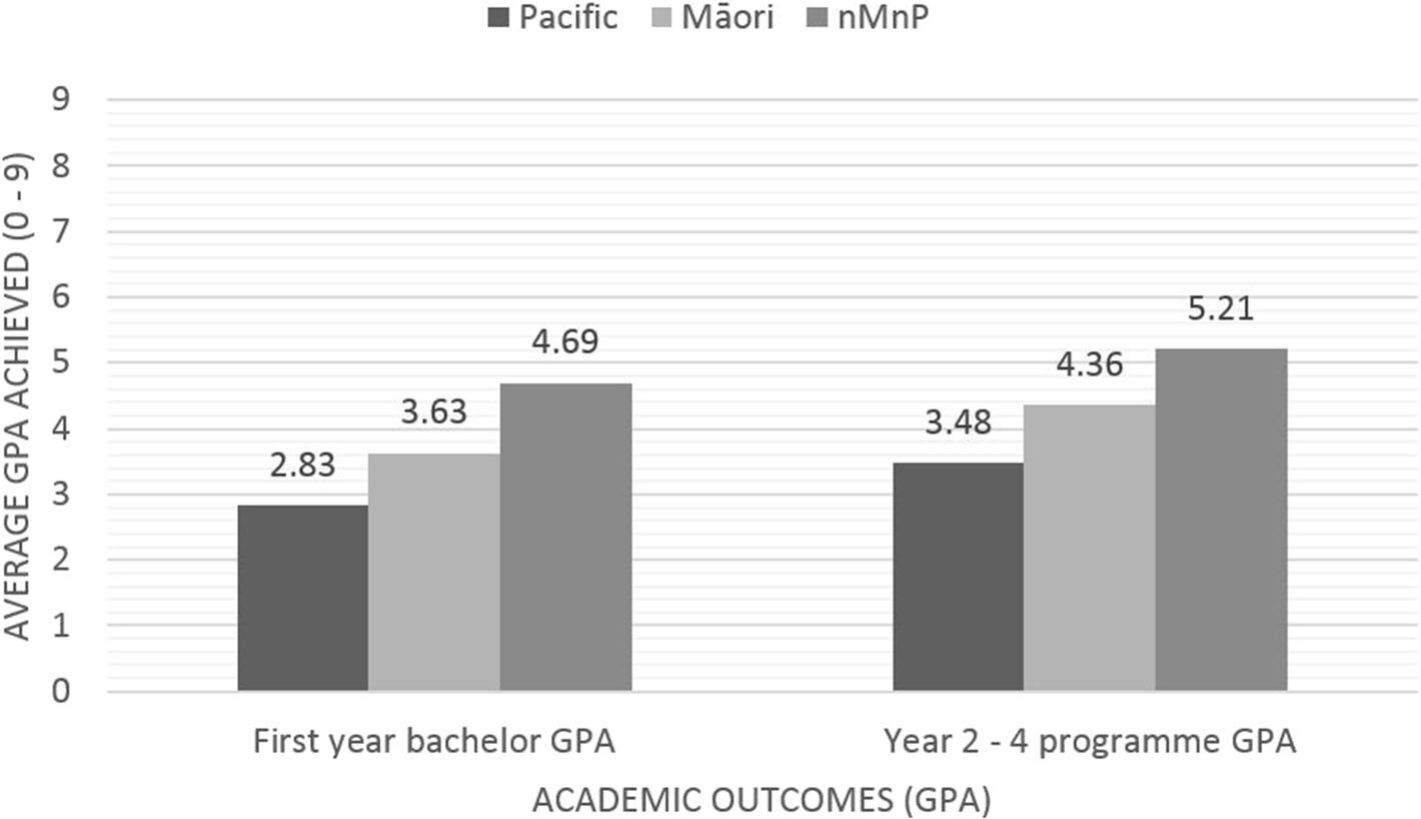
Average first year bachelor and Year 2 – 4 programme GPA achieved for Māori, Pacific and nMnP student groupings

Māori or Pacific students were less likely to have passed all courses in their first year of bachelor study (*p* < 0.0001, 61 % for Māori, *p* < 0.0001, 41 % for Pacific) when compared with nMnP students (78 %), and had an average first year bachelor GPA that was significantly lower (mean GPA = 3.63, SD = 1.71, *p* < 0.0001 for Māori, mean GPA = 2.83, SD = 1.64, *p* < 0.0001 for Pacific) when compared to nMnP students (mean GPA = 4.69, SD = 1.94) (Table 3, Figs. 3 and 4).

**Table 3:** Academic outcomes for Māori, Pacific and non-Māori non-Pacific students

Academic outcome variables	Ethnic grouping									
	Māori (*n* = 150)			Pacific (*n* = 257)			nMnP (*n* = 2279)		Total (*n* = 2686)	
*Categorical variables*	*n*	*%*	*P value*	*n*	*%*	*P value*	*n*	*%*	*n*	*%*
First year bachelors passed all			<0.0001			<0.0001		ref.		
No	59	39.3		152	59.1		492	21.6	703	26.2
Yes	91	60.7		105	40.9		1786	78.4	1982	73.8
*Missing*	0	0.0		0	0.0		1	0.0	1	0.0
Programme passed all			<0.0001			<0.0001		ref.		
No	63	42.0		155	60.3		538	23.6	756	28.2
Yes	86	57.3		102	39.7		1741	76.4	1929	71.8
*Missing*	1	0.7		0	0.0		0	0.0	1	0.0
Graduated FMHS			0.0078			0.0023		ref.		
No	44	29.3		73	28.4		461	20.2	578	21.5
Yes	106	70.7		184	71.6		1818	79.8	2108	78.5
Graduated intended programme			0.0005			0.0007		ref.		
No	51	34.0		80	31.1		496	21.8	627	23.3
Yes	99	66.0		177	68.9		1783	78.2	2059	76.7
Graduated in minimum time^b^			0.0473			<0.0001		ref.		
No	21	21.2		52	29.4		250	14.0	323	15.7
Yes	78	78.8		125	70.6		1533	86.0	1736	84.3
Composite Outcome			0.0002			<.0001		ref.		
Optimal completion	14	9.3		6	2.3		450	19.7	470	17.5
Suboptimal completion high	72	48.0		110	42.8		1193	52.3	1375	51.2
Suboptimal completion low	20	13.3		68	26.5		175	7.7	263	9.8
Non-completion	44	29.3		73	28.4		461	20.2	578	21.5
Programme graduated from^a^			–			–				
BHSc	50	49.0		113	62.1		337	18.6	500	23.9
BNurs	28	27.4		42	23.1		656	36.2	726	34.6
BPharm	24	23.5		27	14.8		818	45.2	869	41.5
*Continuous variables*	*Mean*	*SD*	*P value*	*Mean*	*SD*	*P value*	*Mean*	*SD*	*Mean*	*SD*
First year bachelor GPA	3.63	1.71	<0.0001	2.83	1.64	<0.0001	4.69	1.94	4.45	1.99
Year 2 – 4 programme GPA	4.36	1.90	<0.0001	3.48	1.82	<0.0001	5.21	1.69	5.00	1.79
Year 1 – 4 programme GPA	4.05	1.63	–	3.21	1.56	–	4.95	1.59	4.73	1.68

^a^Students may be double counted if they enrolled in more than one programme. Results are presented for those variables that were tested for significant differences between ethnic groupings (the reference group of comparison is nMnP)

^b^Only calculated for those graduated intended programme

### Programme outcomes

A higher proportion of nMnP students (80 %) graduated from the FMHS compared to 71 % of Māori (*p* = 0.0078) and 72 % of Pacific (*p* = 0.0023) students. A lower proportion of Māori (66 %) and Pacific (69 %) students graduated from their intended programme (i.e. the programme they originally enrolled in) when compared to 78 % of nMnP students (Table 3, Figs. 3 and 4). One in five nMnP students (20 %) had not completed an FMHS programme. Of those that completed an FMHS programme, the majority of Māori (49 %) and Pacific (62 %) students had completed the BHSc programme; 45 % of nMnP students completed the BPharm. Eighty-six percent of nMnP graduates from intended programme had completed their programmes within the minimum time compared to 79 % of Māori students and 71 % of Pacific students (Table 3). The proportion of non-Māori non-Pacific students who passed all courses from year 2 onwards (at first attempt) was 76 % compared to 57 % for Māori and 40 % for Pacific students. The average GPA from year 2 to completion was significantly lower for Māori (mean = 4.36, *p* < 0.0001) and Pacific (mean = 3.48, *p* < 0.0001) students compared to nMnP students (mean = 5.21). When combining the GPA gained across all years of bachelor level study (year 1 – year 3 (BNurs/BHSc) or 4 (BPharm), Māori students had an average GPA of 4.05 (equating to a B- grade), Pacific students had an average GPA of 3.21 (equating to a C+ grade), and nMnP students had an average GPA of 4.95 (equating to B grade).

### Composite graduation outcome

**Fig. 5:**
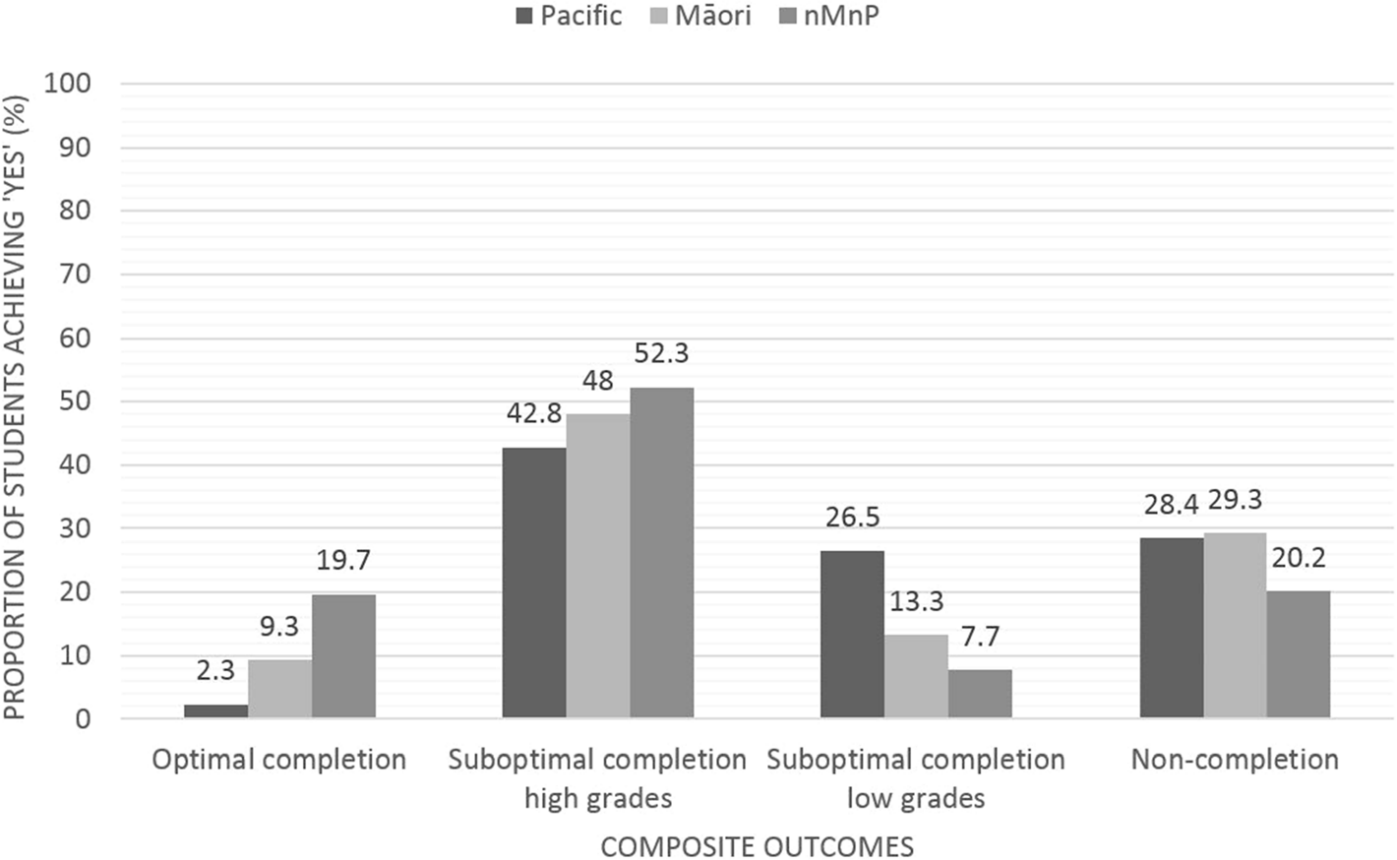
Proportion of students achieving composite completion outcomes by ethnic grouping

For the composite graduation outcome, clear disparities were evident. Whilst 20 % of nMnP students achieved optimal completion (e.g. graduating from intended programme in the minimal time with an A grade average), less than 10 % of Māori and less than 3 % of Pacific students achieved this outcome. Approximately half of Māori (48 %) and nMnP (52 %) students achieved suboptimal completion with high grades compared to only two fifths (43 %) of Pacific students. Eight percent of nMnP students gained suboptimal programme completion with low grades compared to 13 % of Māori and 26 % of Pacific students. A large proportion of both Māori and Pacific cohorts for the composite graduation outcome had not completed a Bachelor level programme within FMHS, with nearly one third being categorised as non-completion (Table 3, Fig. 5).

## Discussion

The findings of this research identify fundamental differences between student ethnic group cohorts. For example, Māori and Pacific students are more likely to have attended lower decile schools, to gain admission via bridging foundation programmes, and to achieve lower secondary school results including lower average credits in science subjects, and Māori students are more likely to be older and have attended school outside Auckland, when compared with nMnP students. The finding that Māori students are slightly older at admission is consistent with literature in this area [43] and is likely to reflect participation in bridging foundation programmes or other pathways prior to entry that increase the amount of time between leaving school and bachelor level programme admission.

### Know your cohort - acknowledging fundamental differences between student cohorts

These research findings indicate that each student ethnic grouping (in particular Māori and Pacific students) is likely to experience a different mix of barriers to academic success depending on the tertiary environment and its responsiveness to such socio-demographic factors [11, 25, 43]. Institutions therefore need to consider the contextual realities of all students they serve and ensure delivery of admission processes, programme content and institutional environments in a way that aims to address such barriers in a comprehensive manner [9, 10]. For example, the FMHS should ensure targeted accommodation support is provided for Māori students given that 42 % attended school outside the Auckland region and will relocate for study purposes compared to 15 % of the total cohort. In addition, with Māori and Pacific student ethnic groupings making up 15.15 % of the total cohort in this study, some would argue that the number of Māori and Pacific teaching staff within the faculty should similarly reflect the student body makeup.

Detailed descriptions of each of the Māori, Pacific and nMnP student ethnic groupings are presented in a way that demonstrates the magnitude of differences between these groupings. These data provide valuable information that extends beyond simply reporting indigenous and/or minority student enrolment numbers [44, 45] to describe pre-tertiary socio-demographic and academic achievement characteristics for each ethnic grouping [2]. Specific values (e.g. average NCEA Rank score) and proportions (e.g. 82 % of Pacific students attended school in Auckland) are presented that were previously unknown for this student cohort. While some national data are available that show rates of achievement of University Entrance by ethnic group for New Zealand school leavers, higher or more detailed academic data such as average NCEA Rank score or subject results for school leavers are not routinely provided or published [46]. It is therefore difficult to know whether the findings of this research align with national data. The findings of this research subsequently provide novel and useful data that can inform secondary and tertiary education sectors.

### Lifting above expectations – what academic preparation for health study really means

The findings of this study show inadequate academic preparation and secondary school achievement for Māori and Pacific students prior to admission. These findings align with Engler (2010b) who found that Māori and Pacific students had lower levels of achievement in NCEA, and therefore, were less well prepared for tertiary study [47]. Whilst inadequate academic preparation for tertiary study generally has been acknowledged previously [10, 25, 26, 47–49], entry into tertiary health professional programmes requires higher prerequisite pre-tertiary achievement than other tertiary education programmes [50–52]. Secondary school students are taught to aim for achieving University Entrance (as the minimum requirement for tertiary education entry) [48]; however, FMHS programmes require secondary school qualifications that far exceed this level of education [50]. Secondary schools often fail to produce a cohort of Māori and Pacific students that a) are able to meet these high academic prerequisites and b) include sufficient numbers for selection [2, 48, 53, 54].

The extent to which these high entry requirements impact on the characteristics of the student cohort chosen for admission are demonstrated by the low number of nMnP students from low decile schools who gain entry into FMHS programmes, indicating that such entry requirements may be privileging (advantaging) those students from medium and high decile schools. This high entry criteria puts added pressure on Māori and Pacific students to meet these additional admission requirements in a secondary education context where their retention until year 13 and participation in science subjects is limited [55].

The context of these research findings necessitates discussion about the meaning of preparation for bachelor level health study. It seems that academic preparation required for FMHS study does not simply involve meeting high prerequisite qualifications, but also obtaining a mixture of specific knowledge, skills, and experiences that boost readiness for bachelor level study [2]. This includes a combination of factors associated with tertiary learning environments in general (e.g. knowledge of course content, exposure to learning environments, readiness for student life), and insider knowledge specific to health study contexts (e.g. heavy science content and high workload expectations) [2, 10]. Tertiary institutions often rely on parents and families to share such information through their own past experiences, however Māori and Pacific students are more likely to be the first in their family to have attended university and hence are less likely to have role models or whānau to share this career information with them [43, 48]. A future workforce report by District Health Boards New Zealand noted that “Māori first-generation tertiary students can be faced with greater challenges, as they are settling into an environment with which their whānau is unfamiliar” (p. 9) [43]. This inevitably leaves students reliant on career advisors, some of whom have been noted to take a deficit analysis and provide inadequate career information to indigenous and minority students [56].

It seems there are clear gaps between the level of academic preparation achieved (or not achieved) by secondary schools and the level of performance expected by tertiary institutions [57, 58]. It is therefore important to monitor this issue over time to ensure narrowing of these gaps.

### Patterns of privilege

Given the observed differences in pre-tertiary and admission variables that are thought to impact on academic success, it is not surprising that differences in all of the academic outcomes investigated were demonstrated between the Indigenous and ethnic minority Māori and Pacific, and the majority non-Māori non-Pacific student groupings. Similar *patterns of privilege* (Figs. 3 and 5) were seen across all of the academic outcome measures, whereby higher/better academic outcomes were produced consistently for nMnP students. Deliberate analysis by ethnic grouping within this research has allowed comparison of ethnic groups and exposed markers of racism and privileging of particular ethnic groups over others [59]. Additional analysis that explores how pre-tertiary, admission and potentially tertiary environment factors might explain the differences in academic outcomes between ethnic groupings is needed. This work is currently underway.

### The role of the university

In the context of the pipeline framework for Māori and Pacific health workforce development [10, 60, 61], the scrutiny of responsibility for ensuring student success at secondary school (and preparation for tertiary study) has focussed on the secondary education sector [23, 62]. The tertiary sector has a key role to play in facilitating this success [63, 64]. The current approach of the health faculty has been to set high entry requirements, enabling selection of the most qualified students (in this cohort reflecting high decile schools) from the available pool (although the Māori and Pacific Admission Scheme (MAPAS) admission process has provided an alternative entry pathway for admission through the CertHSc for those Māori and Pacific students not meeting general entry criteria). This approach has been fuelled by the high demand for places within programmes [65, 66]. In the FMHS context, this approach may have led the institution to set the teaching and learning curriculum to a high standard generally thereby limiting the ability of the programmes to meet the needs of students with lower secondary education qualifications (in this cohort reflecting low decile schools). The setting of high entry criteria may be maintaining and facilitating elitism given that those students who are more likely to succeed academically are also those more likely to come from higher socioeconomic backgrounds, a trend that has been identified internationally [67].

An international report exploring widening participation in European universities noted that *“There has been no improvement in participation at the most selective universities among the least advantaged young people … and the most advantaged young people are seven times more likely to attend the most selective universities as the most disadvantaged”* (p. 5) [67]. Social accountability however demands that widening access to health programmes and therefore increasing health workforce diversity is a priority [18, 68]. Given that detailed data reporting is rarely broken down by ethnic group, opportunity to monitor and critique the institution has also been limited. These findings highlight the need for tertiary institutions to critique the way in which they select and admit all students for tertiary health programmes [18, 66]. Recent research into the MAPAS equity-targeted admissions process focused on identifying the best starting point for academic success for Māori and Pacific students may provide an exemplar for wider institutional use [2]. Social accountability obligations of tertiary institutions demand a greater responsibility to reach out to primary and secondary education sectors and facilitate change [18]. In light of secondary education sector failures, tertiary institutions need to acknowledge their responsibility to assist in improving retention and academic achievement for Māori and Pacific students at secondary school, and make institutional change that reflects the realistic needs, skills and realities of the diverse body of applicants. Whilst support programmes for students are beneficial in general, detailed information by ethnic cohort enables identification of areas where support can be targeted to meet specific needs. Recent acknowledgement by the Tertiary Education Commission for low-decile students to be a targeted equity group (and therefore receive additional funding) is promising however the realisation of this prioritisation within the tertiary institution remains a challenge.

### Strengths

This study carried out a quantitative analysis of student data that has not previously been undertaken in a New Zealand context. The value of these research results will be important in both national and international contexts where there are large gaps data reporting in this level of detail that compares dominant to non-dominant ethnic groups. Additionally, in the New Zealand context, this research provides clear accounts of the gaps between secondary school achievement and tertiary education expectations that can be measured and monitored on an ongoing basis. The dataset created for this research is also valuable in itself given that other related research can be completed using the data used for this study. The use of Kaupapa Māori methodology, informed by Pasifika methodology, is a particular strength of the project as it foregrounds Māori and Pacific worldviews and realities and allows data analysis in a way that may not otherwise have been completed [34, 37].

### Limitations

This study was limited by the available data that are routinely collected by the University and the way in which they are collected [40]. Specifically, the way in which information is collected within the SSO University central database limits the ability to analyse and report on student data by ethnicity in various ways. Availability of data also limited the inclusion of the medical and optometry programmes within this study and additional research is required that includes these programmes in order to present findings across the entire FMHS. This research is currently underway. The scope of available data also limited the ability to analyse other ‘unmeasured’ factors that may have predicted success in this cohort group. For example, institutional factors are not routinely measured; rather, data collection has an overwhelming focus at the level of the individual student as opposed to the institution. The ability to accurately measure socioeconomic status was also limited by available data. Although student home address was available and could have been matched to the New Zealand Census mesh block data that represents deprivation by area of residence (available from Statistics New Zealand), as has been done in previous studies [69], the advisory group acknowledged that this method may have been less reliable given students often use a ‘temporary’ ‘in-term’ address (e.g. student hostel) whilst studying that does not match their ‘home’ location and associated socioeconomic background. Hence, the study used school decile as a proxy for socioeconomic status and acknowledges that this may limit the study findings [47]. Whilst this study presents valuable descriptive summary data for each of the three ethnic groupings, additional multiple regression analysis may provide additional understanding of the effect predictor variables have on each of the academic outcomes. This work is currently underway.

## Conclusions

Institutions need to identify and understand the realities and challenges faced by Māori and Pacific students in pursuit of health careers and ensure provision of tertiary programmes and environments in ways that meet the needs of all students. Detailed analysis of student data by ethnic grouping provides additional information to inform targeted support. Demonstrated disparities in academic outcomes between ethnic groupings show patterns of privilege and should be alarming to tertiary institution and programme staff. If institutions are serious about achieving equitable outcomes for Māori and Pacific students, urgent institutional change is necessary that ensures the unique needs of Māori and Pacific students are met.

## Abbreviations

FMHS: Faculty of Medical and Health Sciences
MAPAS: Māori and Pacific Admission Scheme
NCEA: National Certificate in Educational Achievement
nMnP: Non-Māori non-Pacific
NZ: New Zealand
UoA: The University of Auckland.

## Footnotes

1 This project focusses on the Bachelor of Health Sciences, Pharmacy and Nursing. Medical programme analysis will be completed as a separate project given its’ unique entry criteria. Optometry was not included as the BOpt was located within a different Faculty until recently.

2 Further discussion of Kaupapa Māori principles has been published elsewhere: Kaupapa Māori.com. (2015). Kaupapa Māori. Retrieved from: http://www.kaupapamaori.com/. Smith, G. (1997). The development of Kaupapa Māori: Theory and praxis. Unpublished PhD. The University of Auckland, Auckland.

3 This project acknowledges that ‘Pacific’ is not a recognised ethnic ‘group’, but rather an aggregate category made up of smaller specific ethnic groups (e.g. Tonga, Samoan). Ethnicity categories are therefore herein referred to as ethnic groupings.

4 Direct guaranteed entry to the BHSc programme requires an NCEA Rank Score of 250 combined with at least 18 Level 3 NCEA credits in one subject in each of Table A and Table B.

5 The higher proportion of Māori students who attended school outside of Auckland compared to other ethnic groupings may be reflective of Whakapiki Ake (the Māori student recruitment programme) targeting Māori students both within and outside Auckland to attend FMHS.
